# 雷氏大疣蛛蜘蛛毒素对人肺癌细胞A549增殖的影响

**DOI:** 10.3779/j.issn.1009-3419.2010.10.02

**Published:** 2010-10-20

**Authors:** 增祥 胡, 昱蕾 杜, 全喜 刘, 媛 王, 亮 李

**Affiliations:** 1 050041 石家庄，解放军第260医院内一科 Department of the First Internal Medicine, 260th Hospital of PLA, Shijiazhuang 050041, China; 2 056002 邯郸，河北省邯郸市第一医院胸外科 College of Life Science, Hebei Normal University, Shijiazhuang 050016, China; 3 050011 石家庄，河北师范大学生命科学学院 Department of Thoracic Surgery, the First Hospital of Handan City, Handan 056002, China

**Keywords:** A549细胞, 雷氏大疣蛛蜘蛛毒素, P38MAPK, 抗癌药物, A549 cells, Spider venom, P38MAPK, Anti-cancer drug

## Abstract

**背景与目的:**

雷氏大疣蛛蜘蛛毒素作为新药有可能用于癌症的治疗，本研究旨在探讨雷氏大疣蛛蜘蛛毒素对人肺癌细胞A549的作用及机理。

**方法:**

应用MTT法检测雷氏大疣蛛蜘蛛毒素对人肺癌细胞A549增殖的影响，比色法检测过氧化氢酶活性，改良的硫代巴比妥酸荧光法检测丙二醛含量；流式细胞仪检测细胞凋亡率。采用免疫印迹分析A549细胞中P38MAPK蛋白的表达。

**结果:**

雷氏大疣蛛蜘蛛毒素可抑制A549细胞增殖，使CAT活性和MDA的形成增加，且使P38MAPK的表达较对照组明显增多。

**结论:**

雷氏大疣蛛蜘蛛毒素抑制A549细胞增殖可能与CAT活性和MDA的形成增加以及P38MAPK的表达降低相关。

## Abstract (en)

**Background and objective:**

The spider venom may inspire new drugs to treat cancer. The aim of this study is to investigate the effects and mechanisms of spider venom on lung adenocarcinoma cell A549.

**Methods:**

The proliferation of lung adenocarcinoma A549 cells was detected by MTT. The apoptosis rate was observed with MTT assay and flow cytometer. The activity of catalase was detected by colorimetry. The malondialdehyde (MDA) content was determined by improved thiobarbituric acid fluorometric method. The expression of P38MAPK protein was analyzed with Western blot.

**Results:**

Spider venom can remarkably inhibite the proliferation of lung adenocarcinoma A549 cells, increased activity of catalase and MDA content, down-regulated expression of P38MAPK compared with the control group.

**Conclusion:**

The reduced proliferation of lung adenocarcinoma A549 cells by spider venom is may be associated with the increased of activity of catalase and MDA content and decreased expression of P38MAPK.

肺癌是常见的恶性肿瘤之一，近年肺癌的发病率和死亡率都有明显的增高趋势^[1]^。氧化应激反应与肺癌的发生相关。由于多环芳烃会产生活性氧，以及代谢成毒性中间体，可以通过检测过氧化氢酶（catalase, CAT）活性以反映氧化应激相关的信号。丙二醛（malondialdehyde, MDA）是脂质过氧化物的代谢产物。P38MAPK是MAPK家族的重要成员之一，在、应激状态下，与炎症反应及细胞凋亡关系密切。已有文献报道了蛇毒^[2, 3]^、蜂毒^[4, 5]^和蝎毒^[6]^的抗肿瘤作用。目前尚无关于雷氏大疣蛛蜘蛛毒素对人肺癌细胞A549的作用的报道。因此，本研究旨在通过检测雷氏大疣蛛蜘蛛毒素对人肺癌A549细胞增殖、CAT、MDA及P38MAPK表达的影响，为探讨大疣蛛蜘蛛毒素对人肺癌细胞A549的作用机理，有助于探究雷氏疣蛛蜘蛛毒素的抗肿瘤效应，为其是否有可能成为治疗肺癌的新的多肽类药物提供实验基础。

## 1 材料与方法

### 1.1 实验材料

通过电刺激法采集纯的雷氏大疣蛛雷氏大疣蛛蜘蛛毒素。使用前将雷氏大疣蛛蜘蛛毒素冻干并置于-80 ℃环境中。将雷氏大疣蛛蜘蛛毒素溶于缓冲溶液PBS中并离心（1 000 g, 10 min），去除不溶物质。临时配制不同实验的试验溶液，通过用DMEM稀释原液将毒素调整至最终浓度为0 μg/mL、10 μg/mL、20 μg/mL、40 μg/mL。PBS用作阴性对照。DDP购自Sigma有限公司。

人肺腺癌细胞A549来源于美国ATCC（American Type Culture Collection）。用含10%小牛血清RPMI-1640培养基，其内加入2 mmol/L的谷氨酰胺+0.05 mmol/L 2-巯基乙醇（2ME）+10%胎牛血清。保持细胞浓度为2×10^5^/mL-9×10^5^/mL，在37 ℃、5%CO_2_饱和湿度的条件下培养。甘油作为其冷冻保护剂。2.5 g/L胰酶消化传代。实验时取对数生长期细胞。首先将细胞在10%FCS中培养2天。然后每2天通过离心、洗涤6次且传代的方式收集细胞。

将处理过的雷氏大疣蛛蜘蛛毒素作用到A549细胞。在处理和控制细胞培养24 h后，再将其收获进行分析。本实验中的所有数据至少出自3次独立的实验，均显示出同样的表达模式。

### 1.2 实验方法

#### 1.2.1 MTT法检测雷氏大疣蛛蜘蛛毒素的细胞毒性

细胞增殖抑制实验通过MTT法^[8]^检测。将A549细胞种在96孔板中，100 mL培养基中1×10^4^个细胞。24 h后，用200 mL PBS取代96孔板中的培养基并且加入不同浓度的雷氏大疣蛛蜘蛛毒素（8 μg/mL, 16 μg/mL, 32 μg/mL），DDP作为阳性对照和PBS作为阴性对照。细胞在37 ℃环境下培养48 h。每个孔加入50 mL的MTT溶液。培养4 h后，样品溶于二甲基亚砜（DMSO）中，并用酶标仪检测样品吸光度，测定波长492 nm，参考波长690 nm。OD值表示出试验组和对照组的光密度。重复实验3次。采用线性分析确定IC_50_值。

#### 1.2.2 CAT活性的检测

以过氧化氢为底物，用紫外分光光度法测定其被CAT降解量，间接计算CAT活力。过氧化氢酶降解率（240 nm测量）用来检测雷氏大疣蛛蜘蛛毒素处理细胞和对照组细胞的过氧化氢酶活性的变化^[9]^。

#### 1.2.3 MDA含量的检测

脂质过氧化水平，通过MDA含量来检测，用硫代巴比妥酸（TBA）方法^[10, 11]^。

#### 1.2.4 流式细胞仪检测细胞周期

A549细胞调整为5×10^5^ /mL的细胞浓度。孵育48 h后，用雷氏大疣蛛蜘蛛毒素处理的细胞与未经雷氏大疣蛛蜘蛛毒素处理的细胞进行平行对照。细胞用70%乙醇收集并悬浮，4 ℃，1 h。用胰蛋白酶进行收集并用PBS液冲洗3次。将悬浮颗粒置于250 mL PBS和100 mL Annexin V/PI溶液中。在避光室温情况下碘化染色30 min，细胞悬浮液通过流式细胞仪（BD公司，美国）进行检测。测定细胞周期，观察G_1_期、G_2_期、S期各期细胞所占的百分比，观察是否存在凋亡峰。通过WinMDI 2.5版本软件（TSRI流式细胞术）来分析。

#### 1.2.5 Western blot检测P38MAPK的表达^[12]^

通过缓冲液裂解制备A549全细胞裂解液，缓冲液中含有1%Nonidet P-40、乙磺酸50 mmol/L（pH7.5）、氯化钠100 mmol/L，EDTA 2 mmol/L、焦磷酸缓冲液1 mmol/L、原钒酸钠10 mmol /L、苯甲基磺酰氟1 mmol/L以及氟化钠100 mmol/ L。相同浓度的裂解液与十二烷基硫酸钠反应-10%聚丙烯酰胺凝胶电泳，并用转移缓冲液（Tris 25 mmol/L，甘氨酸192 mmol/L，甲醇10%[v/v]）将其转移到PVDF膜上。PVDF膜用Tris缓冲液（TBS: Tris 0.5 mol/L pH7.6, Nacl 0.15 mol/L）冲洗，并用TBS-5%牛血清白蛋白（BSA）在室温下过夜，含有抗体的TBS-5%BSA孵育。ECL化学发光试剂盒（Kirkegaard & Perry Labo-ratories）显色进行检测P38MAPK的表达。

### 1.3 统计学分析

采用SPSS 10.0统计软件进行分析，3次独立实验的数据用Mean±SD表示，组间比较采用χ^2^检验。以*P* < 0.05为有统计学差异。

## 2 结果

### 2.1 雷氏大疣蛛蜘蛛毒素抑制A549细胞增殖

通过细胞生长曲线的回归方程算出24 h雷氏大疣蛛蜘蛛毒素对A549细胞的IC_50_为12 μg/mL。随着浓度范围从8 μg/mL到32 μg/mL，抑瘤率（growth inhibitory rate, GIR）呈现明显的浓度依赖性（图 1）。

**1:**
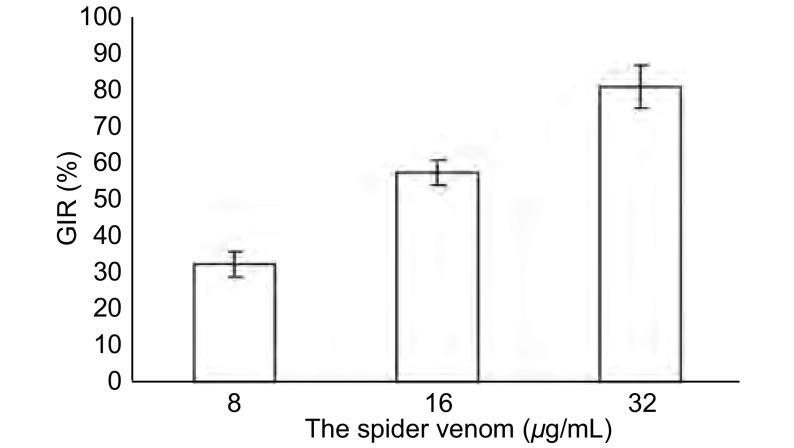
雷氏大疣蛛蜘蛛毒素抑制A549细胞增殖 The inhibition of A549 cells proliferation by spider venom

### 2.2 雷氏大疣蛛蜘蛛毒素对A549细胞中CAT活性和MAD含量的影响

在雷氏大疣蛛蜘蛛毒素作用的细胞中检测到CAT活性和MDA的形成增加（图 2，图 3），但无浓度依赖性（*P* > 0.05）。可见，雷氏大疣蛛蜘蛛毒素参与氧化应激可能是通过增加CAT活性和MAD含量，从而引起对A549细胞的毒性作用。

**2:**
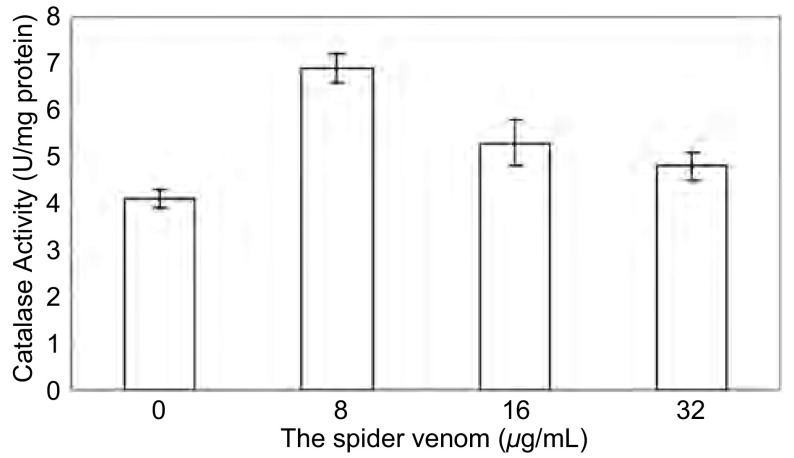
雷氏大疣蛛蜘蛛毒素增加A549细胞CAT活性 Spider venom increased activity of CAT in A549 cell

**3:**
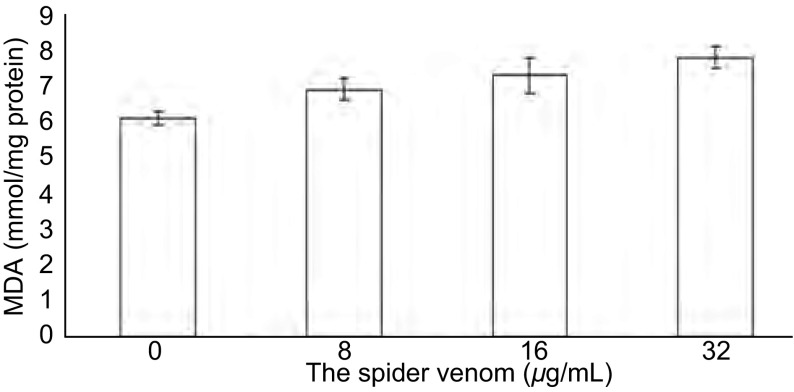
雷氏大疣蛛蜘蛛毒素增加A549细胞MAD含量 Spider venom increased MAD content in A549 cell

### 2.3 雷氏大疣蛛蜘蛛毒素诱导的G_2_/M期细胞周期阻滞

如图 4中所示，细胞周期在48 h周期中连续进行。随着雷氏大疣蛛蜘蛛毒素作用时间的延长，与阴性对照组相比，细胞的G_1_期细胞比例明显减少，S期细胞减少，G_2_期细胞明显增加，细胞阻滞在G_2_/M期。

**4:**
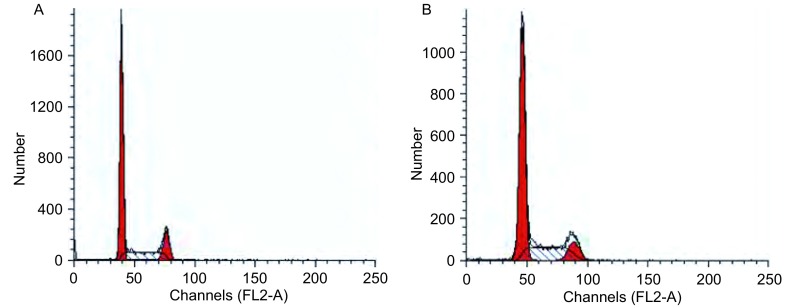
雷氏大疣蛛蜘蛛毒素诱导的G_2_/M期细胞周期阻滞。A：雷氏大疣蛛蜘蛛毒素作用A549细胞前；B：雷氏大疣蛛蜘蛛毒素作用A549细胞后。 The cell cycle distribution of A549 cells by the spider venom. A: before treated by the spider venom; B: after treated by the spider venom.

### 2.4 雷氏大疣蛛蜘蛛毒素对A549细胞中P38MAPK蛋白表达的影响

由图 5可见，随着雷氏大疣蛛蜘蛛毒素浓度的增加，A549细胞中P38MAPK蛋白表达逐渐降低。可见，雷氏大疣蛛蜘蛛毒素抑制A549细胞增殖可能与P38MAPK激酶表达含量降低有关。

**5:**
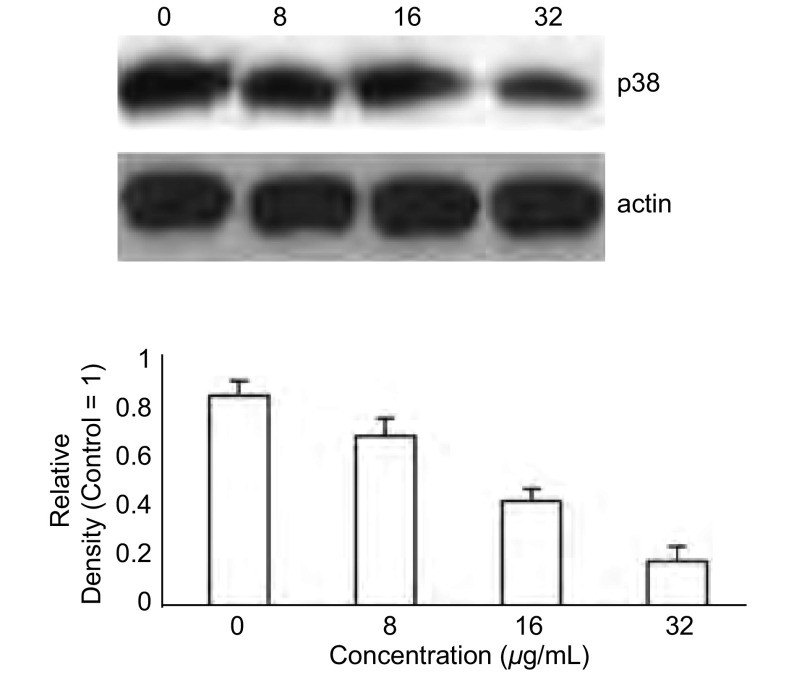
雷氏大疣蛛蜘蛛毒素减少A549细胞中P38MAPK蛋白表达 Spider venom reduced expression of P38MAPK in A549 cells

## 3 讨论

中国是一个肺癌高发的危险区。原发性肺癌治疗仍然困难，依赖于基础医学研究成果。最近的研究^[13, 14]^结果表明，细胞凋亡和周期的改变与肿瘤的发生、进展和转移密切相关。

本研究结果表明，在A549细胞经雷氏大疣蛛蜘蛛毒素24 h作用后，浓度依赖性诱导凋亡。通过MTT法测定雷氏大疣蛛蜘蛛毒素在0 μg/mL、8 μg/mL、16 μg/mL和32 μg/mL剂量时显著抑制了A549细胞增殖。在雷氏大疣蛛蜘蛛毒素作用的细胞中检测到CAT活性和MDA的形成增加，但无浓度依赖性。随着雷氏大疣蛛蜘蛛毒素作用时间的延长，对细胞生长的抑制作用可能是由于G_2_/M期细胞周期的阻滞，并出现一明显的凋亡峰。雷氏大疣蛛蜘蛛毒素可能通过抑制P38MAPK的表达发挥其诱导凋亡作用。据报道MAPK激酶特别是P38蛋白激酶在双向调节细胞周期和细胞凋亡的途径上发挥重要作用^[15]^。P38MAPK激酶对细胞周期调控和细胞凋亡的影响符合这些结论^[16]^。P38MAPK蛋白表达的改变也显示了经雷氏大疣蛛蜘蛛毒素作用后的细胞周期调节表达的模式，意味着雷氏大疣蛛蜘蛛毒素有可能作为一个化学预防药物对肺癌进行治疗和预防。

令人感兴趣的是，P38MAPK表达的减少是由雷氏大疣蛛蜘蛛毒素引起的，但是整个细胞周期的上下游各种蛋白的协调和结合尚未被完全检测。因此，其详尽机理有待在今后的细胞周期调控中继续探索。
